# Effects of opioid-free propofol or remimazolam balanced anesthesia on hypoxemia incidence in patients with obesity during gastrointestinal endoscopy: A prospective, randomized clinical trial

**DOI:** 10.3389/fmed.2023.1124743

**Published:** 2023-03-22

**Authors:** Keyao Zhang, Yuan Bao, Xue Han, Wenshan Zhai, Yi Yang, Meng Luo, Fang Gao

**Affiliations:** ^1^Jiangsu Province Key Laboratory of Anesthesiology, Xuzhou Medical University, Xuzhou, Jiangsu, China; ^2^Department of Anesthesiology, The Affiliated Hospital of Xuzhou Medical University, Xuzhou, Jiangsu, China; ^3^Department of Gaoxin Operating Room, The Affiliated Lianyungang Hospital of Xuzhou Medical University, Jiangsu, China; ^4^Department of Anesthesiology, Suining County People’s Hospital, Xuzhou, Jiangsu, China

**Keywords:** propofol, obesity, ketamine, deep sedation, remimazolam, hypoxemia

## Abstract

There are presently no consensuses on the optimal sedation strategy for obese patients during gastrointestinal endoscopy. This study aim to explore the effects of opioid-free propofol or remimazolam balanced anesthesia on hypoxemia incidence in patients with obesity. A total of 264 patients were randomized to remimazolam + esketamine group (group R) or propofol + esketamine group (group P). Anesthesia in group P was administrated by propofol, esketamine and in group R by remimazolam, esketamine. The primary outcome was incidence of hypoxemia. Secondary outcomes were the time to loss of consciousness (LoC) and to recovery and the incidence of intraoperative and postoperative adverse reactions. We found the incidence of mild hypoxemia in group R was similar to that in group P (14.2% vs. 11.5%, *p* = 0.396). The incidence of severe hypoxemia in group R was significantly lower than Group P (4.2% vs. 9.2%, *p* = 0.019). The time to LoC in group R was longer than group P [Median (interquartile range, IQR): 53 s (45 to 61) vs. 50 s (42 to 54), *p* = 0.001]. The time to recovery from anesthesia in group R was less than group P [Median (IQR): 48 min (41 to 58) vs. 55.5 min (46 to 67), *p*<0.001]. There was no significant difference in the incidence of adverse events (*p* > 0.05 for all). We concluded that compared with propofol combined with esketamine, remimazolam combined with esketamine can reduce the incidence of severe hypoxemia during gastrointestinal endoscopy in obese patients.

**Clinical Trial Registration:** www.chictr.org.cn, Identifier: ChiCTR2200065575.

## 1. Introduction

To eliminate discomforts from mechanical stimulation, gastrointestinal endoscopy procedures are usually performed under deep sedation. Propofol combined with short-acting opioids is currently the preferred anesthetic strategy for endoscopy, due to its reliable sedative and analgesic effects. A large observational study showed that the incidence of hypoxemia with upper gastrointestinal endoscopy in the general population was approximately 15%, and that up to 23.5% of patients with hypoxemia require at least two or more airway interventions (1). Unsuprisely, the incidence of adverse respiratory events may be higher in obese patients (2). Notebly, Patients with obesity have a higher incidence of hypoxemia under deep sedation because they have a relatively low functional residual capacity and higher closed volume (3, 4). A high prevalence of hypoxemia is associated with interoperative adverse outcomes like brain injury (5, 6). Even though hypoxemia is common in endoscopy, this adverse event may cause tachycardia and coronary ischemia during sedation (7). Thus, administering sedation safely in patients with obesity remains a challenge (8).

Opioid-free balanced anesthesia minimizes opioid-related adverse effects (9). Esketamine is the S-enantiomer of ketamine and it has fewer mental side effects, higher clearance rate and receptor affinity. Current researches suggest that esketamine is a more attractive sedative adjunct to propofol compared with opioids (10–12). Esketamine not only has a synergistic effect on sedation, its use greatly reduces the incidences of respiratory and circulatory depression, mental side effects, and vomiting (13). Nevertheless, the effectiveness of this strategy in populations at high airway management risk remains inconclusive. In addition, propofol has obvious disadvantages so as to limit its use, including a narrow therapeutic window, strong injection pain, highly hepatic active enzyme-dependent metabolism, and potential protein allergens (14).

Researches have confirmed that remimazolam, a new type of benzodiazepine, has a stronger and faster sedative effect compared with midazolam, and causes less respiratory and circulatory depression (15, 16). However, its sedative safety and efficacy in those with obesity has not been fully evaluated. The purpose herein was to determine whether remimazolam combined with esketamine is safer and more effective than propofol in patients with obesity. This single-center randomized controlled trial assessed patients at risk of hypoxemia, to explore the effects of these two opioid-free balanced anesthesia strategies on the incidence of hypoxemia in patients with obesity during sedation for endoscopy.

## 2. Materials and methods

### 2.1. Study design

This prospective, randomized, double-blind, controlled trial was approved by the Ethics Committee of the Affiliated Hospital of Xuzhou Medical University (XYFY2022-KL291-02) and was registered at the Chinese Clinical Trial Registry (ChiCTR2200065575). This study strictly followed the guidelines of the Declaration of Helsinki. This study was conducted at the Gastrointestinal Endoscopy Center of the Affiliated Hospital of Xuzhou Medical University, which is the largest gastrointestinal endoscopy center in Xuzhou, Jiangsu Province, China. Nearly 1,000 gastrointestinal endoscopy procedures are performed monthly in this center’s operating room by experienced gastroenterologists and nursing teams. All patients signed informed consent before received allocated intervention.

### 2.2. Participants, randomization, and blinding

Patients scheduled for gastrointestinal endoscopy were eligible to participate if they were aged 18 to 65 years, had a body mass index (BMI) between 30 and 40 kg/m^2^, had American Society of Anesthesiologists Physical Status (ASA PS) grade II or III, had no history of cardiac insufficiency (Including left ventricular ejection fraction >55%, the peak velocity of blood flow of diastolic mitral valve: E/A > 1 and the tircuspid annular plane systolic excursion >13 mm) and were able to communicate normally. Exclusion criteria were known allergy to planned medications, severe hypertension, history of myocardial infarction, severe hepatic or renal dysfunction, mental illness, pregnancy, epileptic disorder, elevated intracranial pressure, use of medication that affects the central nervous system, or modified Mallampati class IV. Secondary exclusion criteria were termination of the clinical protocol for any reason and patients request to withdrawal.

All enrolled patients were randomly allocated in a 1: 1 ratio to either propofol + esketamine group (group P) or remimazolam + esketamine group (group R) using the randomization tool at www.randomization.com, with a random block size of six. Randomization was completed by an independent investigator, and the results sealed in an opaque envelope and delivered to the anesthesiologist responsible for sedation. Normally, two researchers who did not know the specific grouping were present during each procedure, to ensure protocol adherence and record intraprocedural data. The researchers were only responsible for instructing anesthesiologist about the protocol before induction of anesthesia. After the completion of the sedation, the anesthesiologist was responsible for informing the researchers about the intraoperative information after the operation, and the researchers recorded it. Anesthesiologist and investigators were not in the same work area. Except for the occurrence of unforeseen serious adverse events during the procedure, investigators were blind to randomization results.

### 2.3. Interventions

Anesthesiologist assessed enrolled patients before the procedure and recorded basic demographic characteristics. They also carefully assessed patient airway safety and screened patients for OSA using the STOP-BANG (SB) questionnaire (17). All patients underwent the same preprocedural preparation, including strict fasting for 6 h. Patients entered the preparation room and a peripheral venous channel was established in the right upper extremity. Next, 500 ml of balanced salt solution was infused at a rate of 250 ml/h. Electrocardiogram and heart rate (HR) monitoring were performed and pulse oxygen saturation (SpO_2_) and mean arterial pressure (MAP) were measured. The pulse oximeter was not placed on the same upper extremity as the blood pressure cuff, to prevent false drops in saturation during cuff inflation. All patients received oxygen *via* nasal cannula at a flow rate of 4 to 6 l/min, and an airflow detector at the tip of the nasal cannula detected respiratory rate. At the beginning of the gastrointestinal endoscopy, patients were positioned in the left lateral decubitus.

According to their adjusted body weight (ideal body weight + 0.4 × [total body weight − ideal body weight]), the two patient groups were intravenously administered a bolus of esketamine solution (5 mg/ml, 0.5 mg/kg, completed within 10 s). After injection, group P received 1 mg/kg of propofol intravenously (10 mg/ml) and group R recieved 0.1 mg/kg of remimazolam intravenously (1 mg/ml). Both groups were injected slowly, at a rate of 0.5 ml/s until the eyelash reflex disappeared. An anesthetist was responsible for appropriate sedative rescue. When patients had swallowing reflex or Modified Observer’s Assessment Alert/Sedation (MOAA/S) score > 2, groups P and R were administered a single bolus of 1 mg/kg propofol and 0.05 mg/kg remimazolam, respectively and repeated the process if necessary. The MOAA/S scale is a modified OAA/S scale which describes sedation level in greater detail, with a score range from 5 to 0. Whether to perform sedation rescue was determined by the anesthesiologist and endoscopist according to the MOAA/S score. Sedation failure was defined as insufficient sedation after the initial dose and up to four additional doses of remimazolam/propofol within 15 min.

**Figure 1:**
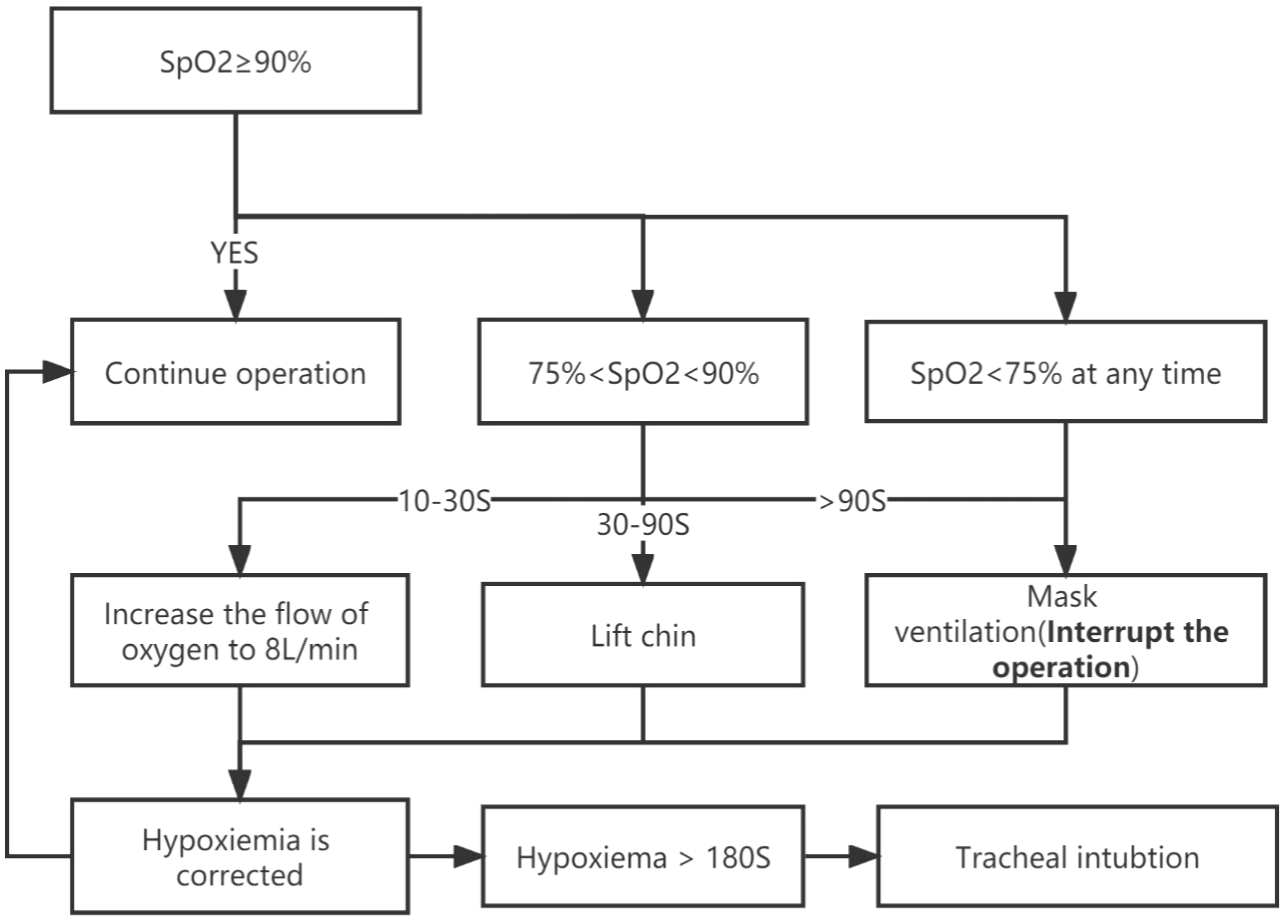
Flowchart of hypoxemia management. SpO_2_ indicates pulse saturation.

During the procedure, HR, SpO_2_, and MAP were continuously monitored. The management of hypoxemia was shown in Figure 1. If an adverse cardiovascular reaction lasted >60 s, the anesthesiologist intervened with appropriate vasoactive drugs. Post-procedure, patients were sent to the post-anesthesia care unit (PACU) for anesthesia recovery monitoring. The modified Aldrete scale was used to assess the quality of patient recovery on five dimensions: respiration, blood pressure, SpO_2_, activity, and consciousness (18). Patients were allowed to leave the PACU when their Aldrete score was ≥9 or equal to their pre-procedure level. The quality of patient recovery and any adverse reactions within 24 h post-procedure (e.g., dizziness, nausea, vomiting, chills, headache, drowsiness) were registered by the postoperative investigators.

### 2.4. Outcomes

For ethical and safety reasons, we chose hypoxemia incidence and type in both groups as the primary endpoints (i.e., the primary outcome was SpO_2_ < 90%, regardless of duration). Hypoxemia was further divided into mild (SpO_2_ 75 to 90% for <60 s) and severe (SpO_2_ < 75% at any time, or SpO_2_ 75 to 90% for >60 s). We instructed the team to perform rapid airway intervention (including chin lift, mask ventilation) when SpO_2_ < 90% for ≥10 s to avoid patient harm from prolonged hypoxemia (19). Secondary outcomes were: incidence of adverse cardiovascular event (including bradycardia, tachycardia, hypotension, or hypertension [>20% change from baseline]); incidence of accidental intubations; incidence of sedation failure; and requirement of sedative rescues (Refers to the proportion of patients who received the maximum number of remedial sedation allowed within 15 min of induction). All adverse respiratory and cardiovascular events were defined according to the World SIVA International Sedation Task Force (20). Other outcomes included: time to loss of consciousness (LoC, defined as the time from induction to disappearance of the eyelash reflex); time to leave the PACU; and incidence of any adverse effects during follow-up.

### 2.5. Sample size

We enrolled 20 patients with obesity received sedation strategy of group P in a small preliminary trial, in which we found a total hypoxemia incidence of 28%. In this prospective clinical trial, we assumed that patients in group R would have a 14% incidence of hypoxemia compared with group P. With a one-sided alpha of 2.5% and power of 80%, 129 patients were needed in each group. We increased the sample size to 282 in both groups to accommodate dropouts and study terminations for any reason. Sample size estimates were calculated using PASS version 15.0 (NCSS, Kaysville, UT, United States).

### 2.6. Statistical analysis

Distribution of continuous variables was examined using the Kolmogorov–Smirnov method. Normally distributed continuous variables are expressed as mean ± standard deviation (SD) and were compared using the Student’s *t*-test. If not normally distributed, the Mann–Whitney U method was used and expressed as median and interquartile range (IQR). The Hodges–Lehmann method was used to estimate the median difference (MD) and 95% confidence interval (CI) was used to estimate heterogeneity. Categorical variables are expressed as numbers (%) and were compared using the χ^2^ test or Fisher’s exact probability test, as appropriate. The number to treat (NNT, 1/[event rate in control group − event rate in experimental group]) and 95% CI were used to assess treatment effects on randomized groups. Kaplan–Meier survival analysis and log-rank test were used to evaluate the effects of sedation strategy on induction and recovery times, and to examine possible heterogeneity of different baseline variables by stratification.

We assessed treatment effects with *post hoc* analyses by stratifying binary variables that may influence hypoxemia. Multiple mixed-effects logistic regression was used to explore independent factors for hypoxemia. Stratified variables like age, gender, BMI, ASA PS, anamnesis, and endoscopy type were included in univariate logistic regression models. If the regression model likelihood ratio test (model fit) *p* < 0.100, it was included in the mixed-effects multivariate logistic regression. All included variables were tested for collinearity, and variance inflation factor > 5 indicated statistically significant multicollinearity among variables. Interactions among stratified variables and randomized groups were tested with logistic regression. Confidence interval estimates in the bootstrap method were restricted to the 2.5th and 97.5th percentiles. Unless otherwise indicated, the odds ratio (OR) and its 95% CI values were obtained by replicating a mixed-effects logistic model on a bootstrapped sample of 2,000 iterations. A two-tailed *p* < 0.05 was considered statistically significant. *p* values were not adjusted for multiple testing. Data analysis followed the intention-to-treat principle. SPSS v23.0 (IBM, Armonk, NY, United States) was used for all analyses.

## 3. Results

### 3.1. Baseline characteristics

**Figure 2:**
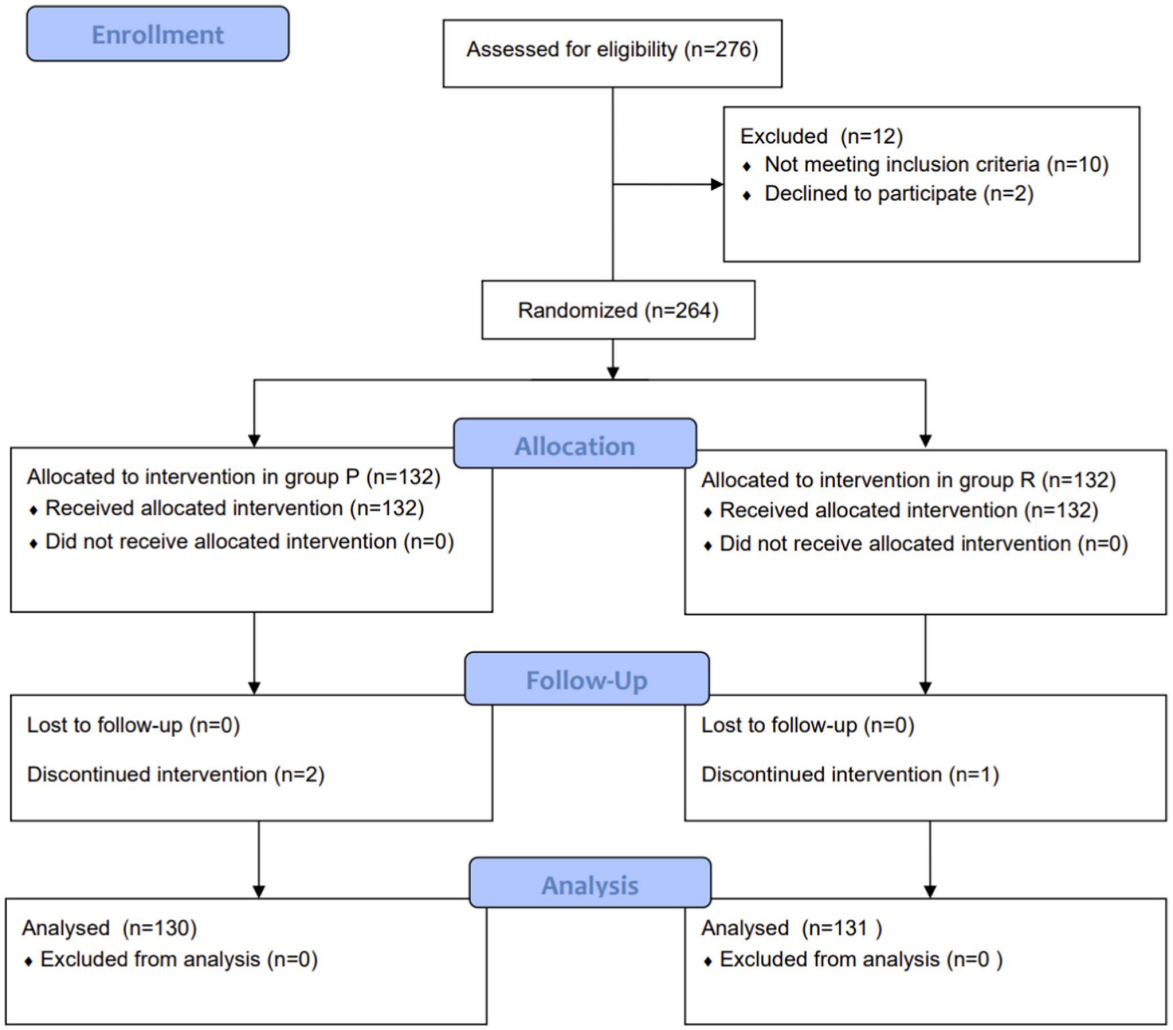
Trial profile.

A total of 261 patients were included in the final analyses: 131 in group R and 130 in group P (Figure 2). Despite the limited sample size, randomization appeared to lead to well-balanced groups. There were no significant between-group differences in age, BMI, comorbidities, or endoscopy type. Respiratory system-related parameters including proportion of patients with neck circumference (NC) > 40 cm, different Mallampati grades, SB score > 5, and lung diseases with different pathophysiological characteristics were also similar between the groups (Table 1).

**Table 1:** Demographic and clinical baseline characteristics.

	Group R (*N* = 131)	Group P (*N* = 130)	*p-*value
Age, yr. (IQR)	45 (39 to 50)	44 (37 to 49)	0.350
Age>45 yr., no. (%)	64 (48.9)	55 (42.3)	0.288
Male, no. (%)	62 (47.3)	77 (59.2)	0.054
Median BMI, kg/m^2^. (IQR)	33.9 (32.4 to 37.2)	34.3 (32.3 to 37.1)	0.984
BMI^a^>35 kg/m^2^, no. (%)	55 (42.0)	60 (46.2)	0.498
Mallampati grades, no. (%)			
I	42 (32.1)	36 (27.7)	0.245
II	71 (54.2)	66 (50.8)	
III	18 (13.7)	28 (21.5)	
*ASA physical status* *^b^* *, no. (%)*			
II	87 (66.4)	78 (60)	0.283
III	44 (33.6)	52 (40)	
neck circumference > 40 cm, no. (%)	60 (45.8)	64 (49.2)	0.579
STOP - BANG Score > 5, no. (%)	63 (48.1)	53 (40.8)	0.234
Hypertension, no. (%)	40 (30.5)	29 (22.3)	0.132
Median SpO_2_ before pre-oxygenation, %. (IQR)	97 (96–98)	97 (96–98)	0.168
Median SpO_2_ at anaesthesia induction, %. (IQR)	99 (98–99)	99 (98–99)	0.528
Ischaemic heart disease^c^, no. (%)	33 (25.2)	39 (30)	0.385
Pulmonary disease, no. (%)			
Asthma	10 (7.6)	7 (5.4)	0.462
Emphysema	5 (3.8)	2 (1.5)	0.255
Restrictive lung disease	2 (1.5)	2 (1.5)	0.994
Diabetes, no. (%)	16 (12.2)	18 (13.8)	0.695
Smoking, no. (%)	38 (29)	34 (26.2)	0.606
Type of gastrointestinal endoscopy, no. (%)			
Upper	65 (49.6)	66 (50.8)	0.908
Lower	39 (29.8)	40 (30.8)	
Combined	27 (20.6)	24 (18.5)	

Values are presented as number and percent (%) for categorical variables and mean (± SD) or median (Inter Quartile Range-IQR) as appropriate for continuous variables.

a BMI (Body mass index), is the weight in kilograms divided by the square of the height in meters.

b ASA (American Society of Anesthesiologists) physical status, is a classification for assessing and communicating preanesthetic medical comorbidities in patients. ASA I is a normally healthy patient, ASA II is a patient with mild systemic disease, ASA III is a patient with severe systemic disease, ASA IV is a patient with persistently life-threatening severe systemic disease, and ASA V is a patient with severe systemic disease. A dying patient who is not expected to survive without surgery; c: patients with at least one or more coronary artery stenosis and > 50% previously identified by coronary angiography, and who had not undergone coronary stenting (Definitively diagnosed ischaemic cardiomyopathy is excluded).

### 3.2. Primary and second outcomes

**Figure 3:**
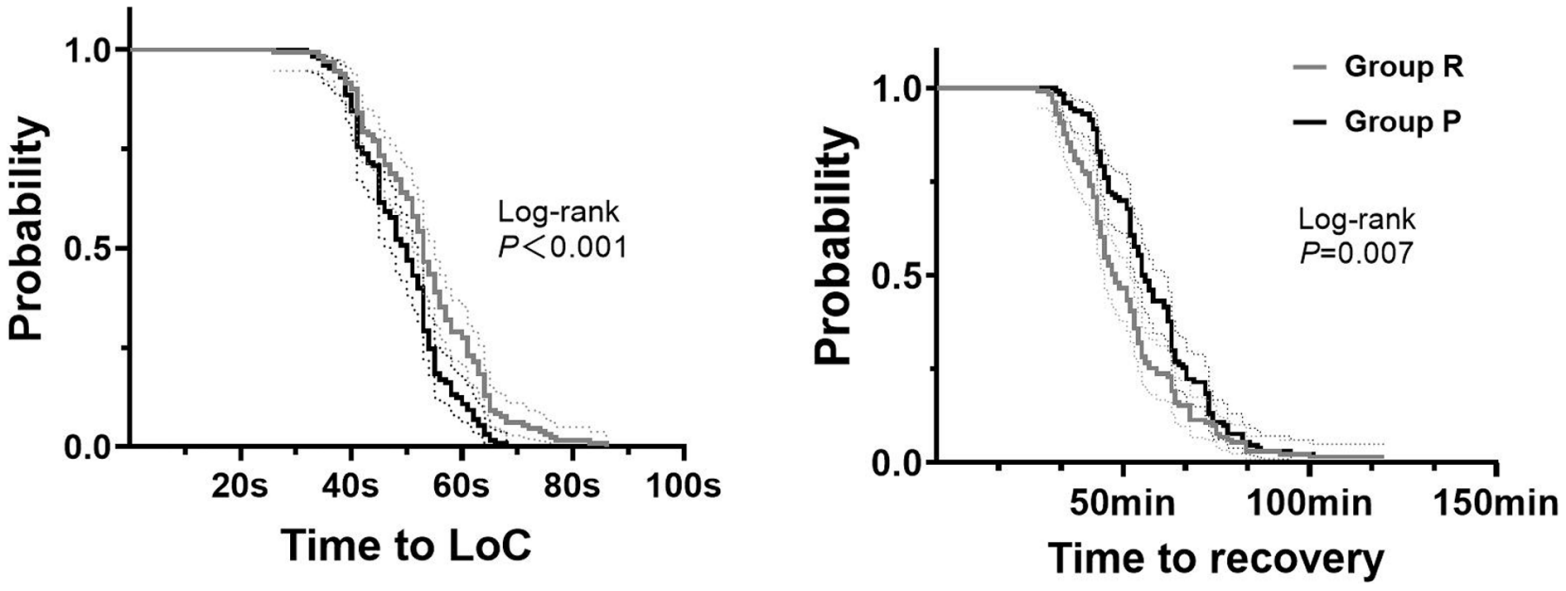
Kaplan–Meier curves for time to induction and recovery. 1. Group R = propofol + esketamine group; Group P = remimazolam + esketamine group; 2. Time to Loc: time to loss of consciousness; 3. The dashed lines represent the 95% confidence interval.

The incidence of mild hypoxemia was similar in groups R and P (14.2% vs. 11.5%, *p* = 0.396). However, the incidence of severe hypoxemia in group R was significantly lower than in group P (4.2% vs. 9.2%, *p* = 0.019, NNT = 10 [95% CI, 5 to 55)]) (Table 2). The time to LoC in group R was longer than in group P (median [IQR]: 53 s [45 to 61] vs. 50 s [42 to 54], *p* = 0.001, MD = 4 s [95% CI, 1 to 6]) (Table 2; Figure 3). There were no accidental endotracheal intubations in either group. Group R required more sedation rescue (19.1% vs. 7.7%, *p* = 0.007, NNT = −9 [95% CI, −31 to −5]) and less airway support than did group P (mask ventilation, 5.3% vs. 13.1%, *p* = 0.031, NNT = 12 [95% CI, 7 to 127]; chin lift, 19.1% vs. 30.8%, *p* = 0.029, NNT = 9 [95% CI, 5 to 78]) (Table 2). There was not a significant difference in the incidence of adverse hemodynamic events during sedation between the groups, including tachycardia, bradycardia, hypertension, and hypotension (*p* > 0.05). There were no significant differences in the incidence of adverse mental events, including headache, hallucinations, and dizziness during recovery (*p* > 0.05). However, the time to recovery from anesthesia in group R was shorter than in group P (median [IQR]: 48 min [41 to 58] vs. 55.5 min [46 to 67], *P*<0.001, MD = −8 min [95% CI, −11 to −4]) (Table 2; Figure 3). Neither time to LoC nor recovery showed significant heterogeneity across subgroups (Supplemental Digital Content 1, Supplementary Table S1). There were no significant interactions between endoscopy type and intervention (Supplemental Digital Content 2, Supplementary Table S2). All adverse events resolved after intervention according to the protocol.

**Table 2:** Procedural characteristics and perioperative outcomes.

	Group R (*N* = 131)	Group P (*N* = 130)	*p*-value	NNT ^#^/MD ^a^.(95% CI)^b^
*Primary outcome measure*				
Mild hypoxemia, no. (%)	37 (14.2)	30 (11.5)	0.396	–
Severe hypoxemia, no. (%)	11 (4.2)	24 (9.2)	0.019	10 (5 to 55)^#^
*Adverse events*				
Bradycardia, no. (%)	10 (7.6)	12 (9.2)	0.642	–
Tachycardia, no. (%)	8 (6.1)	4 (3.1)	0.243	–
Hypotension, no. (%)	15 (11.5)	13(10)	0.705	–
Hypertension, no. (%)	4 (3.1)	1 (0.8)	0.178	–
Dizziness, no. (%)	8 (6.1)	9 (6.9)	0.789	–
Headache, no. (%)	1 (0.8)	2 (1.5)	0.622	–
PONV^c^, no. (%)	2 (1.5)	0	0.498	–
Hallucination, no. (%)	5 (3.8)	3 (2.3)	0.722	–
Chill, no. (%)	0	0	–	–
*Other outcomes*				
Time to LoC^d^, s. (IQR)	53 (45 to 61)	50 (42 to 54)	0.001	4 (1 to 6)^$^
Duration of operation, min. (IQR)	16 (14 to 17.5)	15 (13 to 18)	0.083	1 (0 to 1)^$^
Time to recovery^e^, min. (IQR)	48 (41 to 58)	55.5 (46 to 67)	<0.001	−8 (−11 to −4)^$^
Chin lift, no. (%)	25 (19.1)	40 (30.8)	0.029	9 (5 to 78)^#^
Mask ventilation, no. (%)	7 (5.3)	17 (13.1)	0.031	12 (7 to 127)^#^
Sedation rescue^f^, no. (%)	25 (19.1)	10 (7.7)	0.007	−9 (−31 to −5)^#^
Sedation failure, no. (%)	0	0	–	–
Accidental intubations, no. (%)	0	0		–
Use of Vasoactive drugs, no. (%)	9 (6.9)	11 (8.5)	0.629	–
*Sedative drug total consumption* ^g^				–
Propofol, mg (IQR)	–	134 (120 to 157)	–	–
Remimazolam, mg (IQR)	13.5 (12 to 15)	–	–	–
Esketamine, mg (IQR)	38 (36.5 to 42)	39.5 (36.5 to 44)	0.227	–

Values are presented as number and percent (%) for categorical variables and mean (± SD) or median (Inter Quartile Range-IQR) as appropriate for continuous variables.

# NNT (number needed to treat). Only given if both the upper and lower bounds of the NNT’s 95% confidence interval are positive or negative (to avoid confusing numbers that require treatment and numbers that require harm).

a MD (median difference).

b 95% CI (95% confidence interval).

c PONV (Postoperative nausea and vomiting).

d Time to Loc (time to loss of consciousness), assessed by modified observer’s assessment alert/sedation scale.

e Time to recovery, assessed by modified Aldrete scale, which monitoring from five dimensions: respiration, blood pressure, SpO_2_, activity, and consciousness, and the patients were allowed to leave the PACU when the modified Aldrete scale score ≥ 9.

f refers to patients with up to three additional doses of remimazolam/propofol within the initial dose and 15 min.

g The consumption of propofol and remimazolam was the total dosages required for induction and maintenance.

### 3.3. Exploratory analysis

**Figure 4:**
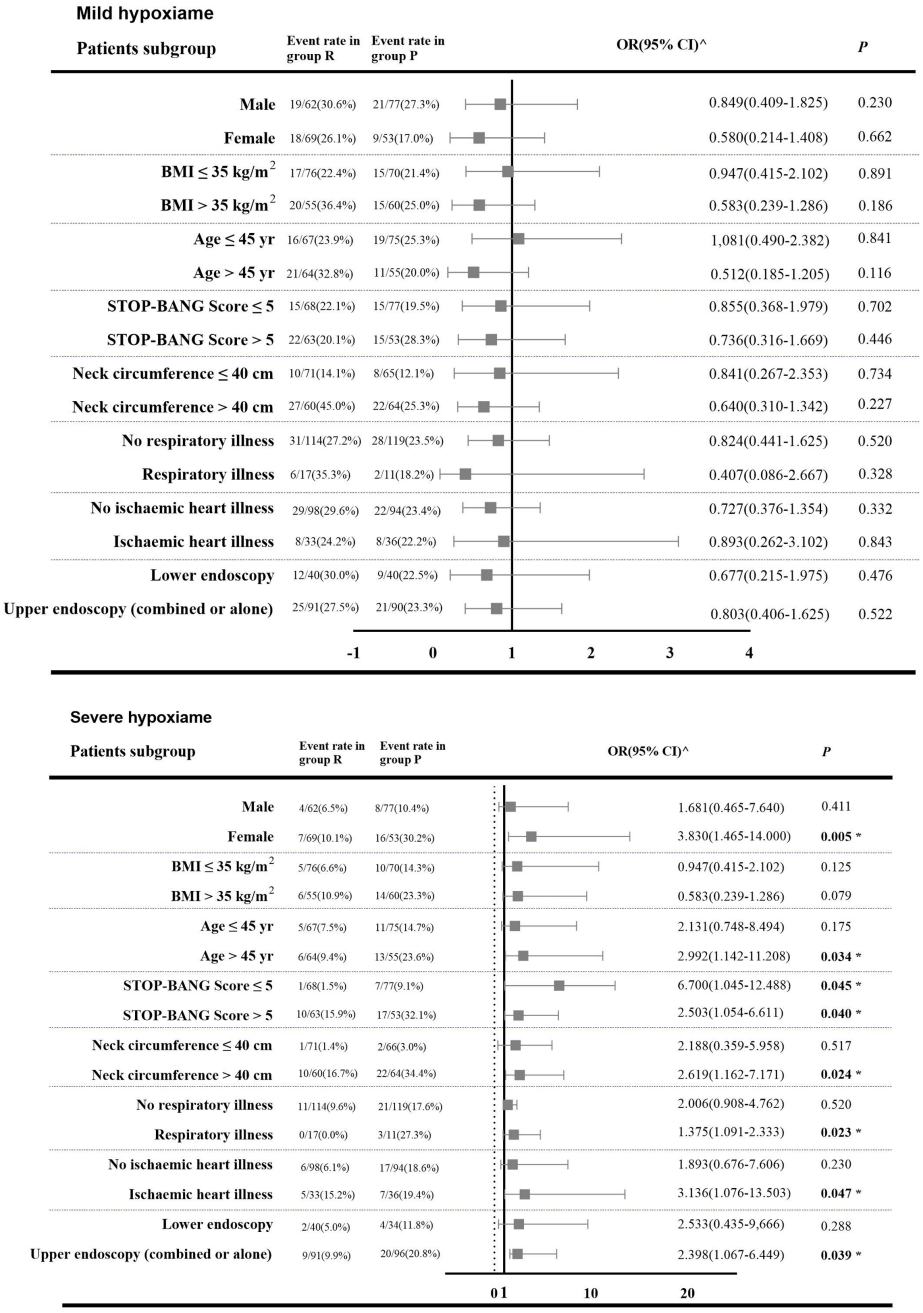
Forest plot showing odds ratiao in the occurrence of hypoxemia in subgroups of patients in both intervention groups. 1. Group R = propofol + esketamine group; Group P = remimazolam + esketamine group; 2. BMI (body mass index) is the weight in kilograms divided by the square of the height in meters; 95% CI (95% confidence interval). 3. ^: The number of outcome events in these patient categories is too small so that bootstrapping and logistic regression are not feasible; OR (95% CI) were obtained from Mantel–Haenszel (MH) estimates (21). 4. The *p*-values of some cases were obtained by the Fisher’s exact test. 5. *: A two-tailed test *p* < 0.05 was considered statistically significant.

The effect of the intervention on incidence of mild hypoxemia was consistent across all subgroups. However, there was heterogeneity in the treatment effect for severe hypoxemia based on gender, age, NC, ischemic heart disease, existence of respiratory comorbidities, and endoscopy type (Figure 4). Univariate logistic regression analysis showed that gender, age, BMI, SB score, NC, and cardiac comorbidities may be independent risk factors for development of hypoxemia (*p* < 0.100). There was no significant interaction between the intervention and specific binary variables (Supplemental Digital Content 2, Supplementary Table S2). Multivariate mixed-effects logistic regression analysis showed that, with patients without obesity or hypoxemia as a reference, in obese patients with mild hypoxemia, NC ≤ 40 cm was a significant independent protective factor (adjusted OR [aOR]: 0.140, [95% CI: 0.071 to 0.275]; *p* < 0.001). Likewise, SB score ≤ 5 (aOR: 0.279, [95% CI: 0.102 to 0.761]; *p* = 0.008), NC ≤ 40 cm (aOR: 0.050, [95% CI: 0.014 to 0.180]; *p* = 0.001), and no history of ischemic heart disease (aOR: 0.342, [95% CI: 0.138 to 0.851]; *p* = 0.029) were also independent protective factors against developing severe hypoxemia (Supplemental Digital Content 3, Supplementary Table S3).

## 4. Discussion

Given increasing demands for safe and comfortable endoscopy, more work needs to be done by anesthesiologists to explore optimal sedation strategies for specific high-risk populations. Increased airway fragility and reduced chest wall compliance make patients with obesity particularly prone to desaturation under traditional opioid-based balanced anesthesia (3). Although high-flow nasal cannula (HFNC) decreases the incidence of desaturation in sedated patients with obesity (22), the high cost of these systems and the training needed for professional practitioners to operate them has limited widespread adoption (22, 23). Thus, lower cost and easier use may facilitate implementation of improved clinical protocols, while maintaining safety. By comparing the effectiveness of two opioid-free balanced anesthesia approaches to painless gastrointestinal endoscopy in patients with obesity, we found that compared with propofol + esketamine, remimazolam + esketamine reduced the incidence of severe hypoxemia. NNT results suggest that using remimazolam + esketamine may prevent severe hypoxemia in 1 in 10 patients with obesity.

Hypoxemia severity depends on both oxygen reserve capacity and degree of respiratory center depression during sedation. High respiratory vulnerability in patients with obesity is mainly attributed to fatty deposits in the soft tissues of the pharyngeal wall and large tongue bodies that are prone to sinking (24). Decreased pharyngeal wall muscle tone and collapse of the upper airway during sedation further exacerbate difficult airway management (24). Guo et al. (25) explored the efficacy of an initial remimazolam bolus of 0.15 mg/kg in gastrointestinal endoscopy sedation in older adults; compared with propofol, this significantly reduced the incidence of hypoxemia. Unlike those with obesity, older adults have low respiratory reserve capacity, high airway sensitivity, increased respiratory center sensitivity, and weakened respiratory protective reflexes. Thus, the protection provided by remimazolam for respiratory center sensitivity may be especially meaningful, partly explaining why the incidence herein of severe hypoxemia in group R was lower than that in group P. Interestingly, propofol has the property of dilating the bronchi in *ex vivo* animal models, under both normal and hypoxic conditions, related to its inhibition of the release of active substances like cabergoline, histamine, and prostaglandins (26). However, the advantages of these properties for patients with obesity and airway diseases remain to be confirmed by extensive mechanistic and clinical studies.

The anesthesia recovery time in group R was shorter than that in group P. While we found no sedation failure in either group, group R required more frequent sedation rescue, similar to findings by Chen et al. (27). Remimazolam can be rapidly hydrolyzed into inactive metabolites by non-organ-dependent metabolism, while pharmacokinetic models indicate that total remimazolam clearance is independent of body weight (28). This may explain the difference in pharmacokinetics and apparent distribution volumes between remimazolam and propofol in patients with obesity. Although obesity increases both fat and muscle, the proportion of adipose tissue is higher, and provides a marked reservoir for high-fat-soluble anesthetics. Although propofol clearance and peripheral compartment volume are significantly higher in patients with morbid obesity (MO), the half-life concentration in these patients is significantly lower than that of patients with standard weights (by ~37.9 to 38.6%), suggesting that the population with MO may be more sensitive to propofol (29). Consequently, increasing the initial dose of remimazolam, for more rapid induction and less rescue sedation, may be feasible in patients with obesity.

The safety of remimazolam for procedural sedation in colonoscopy with the ASA III/IV population was confirmed by Rex et al. in the only open-label clinical study to date (30). Dai et al. (31) also confirmed that when the initial bolus of remimazolam is within 0.2 to 0.4 mg/kg, the time to LoC remains similar to a propofol bolus of 2 mg/kg. Even among populations with hepatic or renal impairment, remimazolam retains predictable pharmacokinetic properties and dosage adjustments are unnecessary (32). Unfortunately, few studies have examined the effects of obesity on the pharmacokinetics of remimazolam in detail. Superior sedation strategies benefit both the anesthesiologist and the patients. Of course, considering the patient’s own interests, we must consider the possible increased cost of group R sedation strategy. However, in busy units, shorter PACU discharge times and higher respiratory protection are clearly valuable. Thus compared to HFNC, which is costly, there is reason to believe that the group R sedation strategy has a huge potential advantage in anesthesia outside the operating room.

Exploratory analyses showed that patients with obesity and ischemic heart disease are more likely to develop severe hypoxemia. Pre-existing cardiac comorbidities increase susceptibility to hypoxemia during gastrointestinal endoscopy because ischemic myocardial tissue is more prone to pump dysfunction, which in turn reduces the oxygen exchange rate between the pulmonary capillaries and alveolus (3, 33). This context may deteriorate when ischemic myocardial tissue is subjected to hypoxia. Thus, there is support for the notion that our group R sedation protocol can be preferentially recommended for patients with these risk factors.

We also found that SB score > 5 and NC > 40 cm are independent risk factors for hypoxemia during sedation in patients with obesity. Logically, patients with BMI > 30 kg/m^2^ should also be among the high-priority patients (24, 33). However, our analyses did not show significant heterogeneity of treatment between patient groups with a BMI of 30 to 40 kg/m^2^, thus these data do not support BMI > 35 kg/m^2^ as independent risk factor for hypoxemia. Low sensitivity of BMI may be attributed to large individual differences in body fat percentage, age, gender, muscle mass, and ethnicity (34). NC measurements has been confirmed to be practical, simple, inexpensive, timesaving, and less invasive than BMI. Kroll et al. (35) have suggested that NC is an accurate tool for assessing/screening obesity across age, gender, and weight categories. However, in females larger NC is more likely to be associated with disproportionately accumulated fat, in contrast to lean tissue, which is a significant contributor to NC in male (36). The proportion of lean tissue in the necks of females may be lower than that of males with otherwise relatively consistent NC; thus, supporting tension for the pharynx of females with obesity would be relatively weaker, increasing their likelihood of upper airway collapse. This may partly explain between-group treatment heterogeneity among females. Further, NC is closely related to the occurrence and development of obstructive sleep apnea (37). Thus for patients with obesity, NC and SB score may be more effective than BMI for assessing the risk of hypoxemia, and are important factors in deciding whether to implement protective sedation strategies.

Several important limitations must be considered when interpreting these results. First, although all patients received low-flow nasal cannula oxygen (4 to 6 l/min) during sedation, FiO_2_ changes were unpredictably influenced by patient-related factors like open mouth (38). Second, we did not include patients with BMI > 40 kg/m^2^ or Mallampati class IV, for ethical and safety reasons, limiting both sample size and generalizability. Third, the specificity of the sample and avoidance of unpredictable risk events meant that dose restrictions in both groups were relatively stringent. Finally, some subgroups lacked sufficient statistical power; further evidence from larger samples and multi-center studies is thus needed, and we did not describe trends in pulse oxygen saturation during sedation in both groups. Besides, the guiding significance of using the bispectral index (BIS) to study remimazolam was not clear; therefore, we had no confidence in using BIS and chose the MOAA/S score instead. Meanwhile, We must admitted that due to the subjective nature of the scale, we did not choose to record the MOSS/A scores in real time. Therefore, we could not completely rule out the clinical outcomes we observed were due to differences in the sedative efficacy of remimazolam and propofol in obese patients.

In summary, we concluded that the sedation strategy of remimazolam combined with esketamine shows a higher safety profile for gastrointestinal endoscopy in patients with obesity compared with propofol combined with esketamine. However, the advantages of this strategy in this and other high-risk populations remains to be confirmed by larger clinical trials.

## Data availability statement

The data analyzed in this study is subject to the following licenses/restrictions: The data associated with the paper are not publicly available but are available from the corresponding author on reasonable request. Requests to access these datasets should be directed to gaofangxz@126.com.

## Ethics statement

This prospective, randomized, double-blind, controlled trial was approved by the Ethics Committee of the Affiliated Hospital of Xuzhou Medical University (XYFY2022-KL291-02) and strictly followed the guidelines of the Declaration of Helsinki.

## Author contributions

KZ: proposing initial conception and methods and writing original manuscript. YB: statistical analysis and editing the manuscript. XH: intraoperative trial protocol supervision and security management. WZ: intraoperative trial protocol supervision. YY: postoperative follow-up and investigation. ML: responsible for patients recruitment and grouping. FG: concept optimization and reviewing manuscript. All authors contributed to the article and approved the submitted version.

## Conflict of interest

The authors declare that the research was conducted in the absence of any commercial or financial relationships that could be construed as a potential conflict of interest.

## Publisher’s note

All claims expressed in this article are solely those of the authors and do not necessarily represent those of their affiliated organizations, or those of the publisher, the editors and the reviewers. Any product that may be evaluated in this article, or claim that may be made by its manufacturer, is not guaranteed or endorsed by the publisher.
