# Supplementary material for: Effects of opioid-free propofol or remimazolam balanced anesthesia on hypoxemia incidence in patients with obesity during gastrointestinal endoscopy: A prospective, randomized clinical trial
Source: Front Med (Lausanne). 2023 Mar 22;10:1124743. doi: 10.3389/fmed.2023.1124743 (PMC10073760; doi:10.3389/fmed.2023.1124743)
Supplement: Supplementary file 1 [file Table_1.docx]

# *Supplemental Digital Content 1*

# Supplementary Tables

***1..1 Time to LoC (timing unit: s )***

**
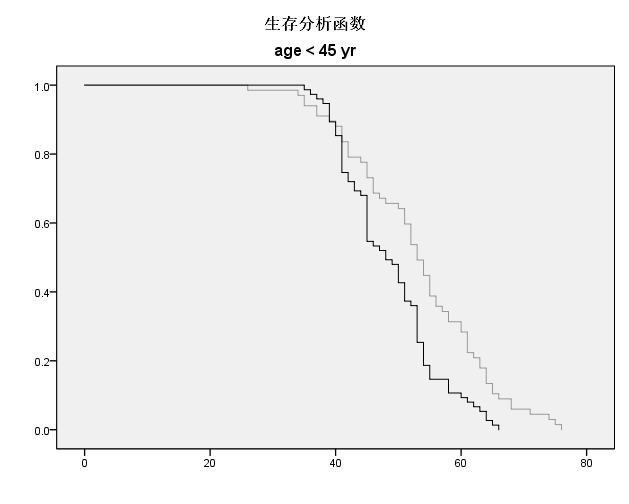

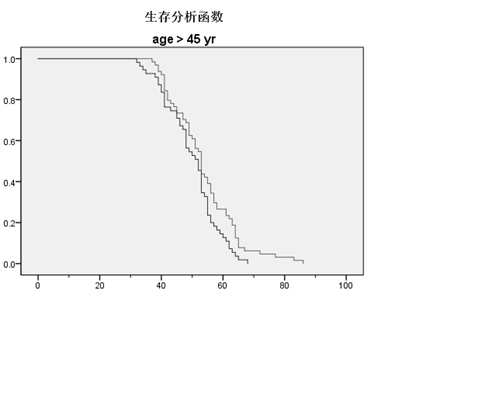
Age**

|  | | Median | 95% CI | | Log Rank (*P*) |
| --- | --- | --- | --- | --- | --- |
|  |  |  | lower | upper |  |
| Age ≤ 45 yr | R group | 53 | 50.594 | 55.406 | 0.001 |
|  | P group | 48 | 45.32 | 50.68 |  |
| Age > 45 yr | R group | 53 | 51.055 | 54.945 | 0.03 |
|  | P group | 52 | 48.984 | 55.016 |  |


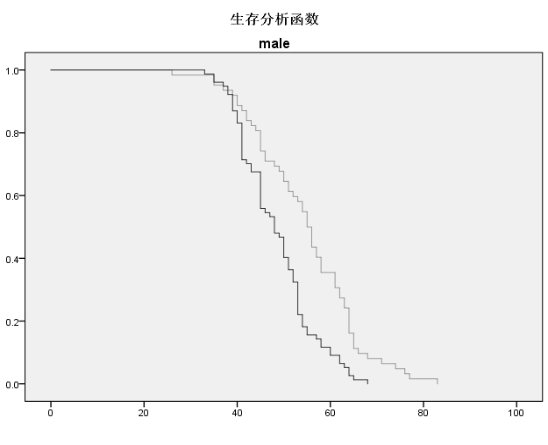
**
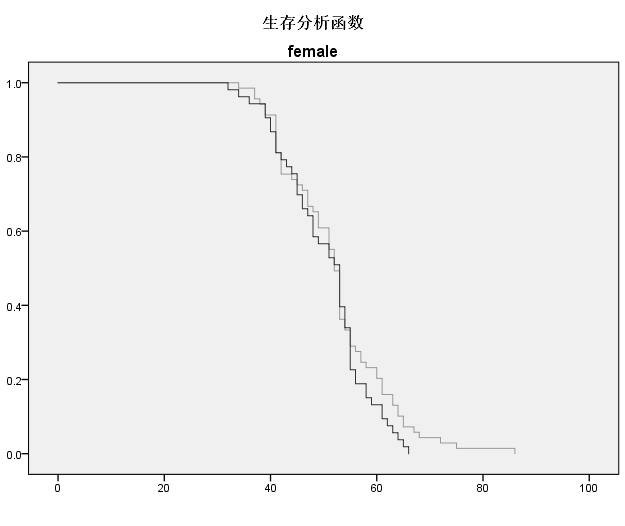
Gender**

|  | | Median | 95% CI | | Log Rank (*P*) |
| --- | --- | --- | --- | --- | --- |
|  |  |  | lower | upper |  |
| Female | R group | 52 | 50.748 | 53.252 | 0.236 |
|  | P group | 53 | 49.898 | 56.102 |  |
| Male | R group | 55 | 52.428 | 57.572 | < 0.001 |
|  | P group | 48 | 44.420 | 51.580 |  |

**BMI** (Body mass index)


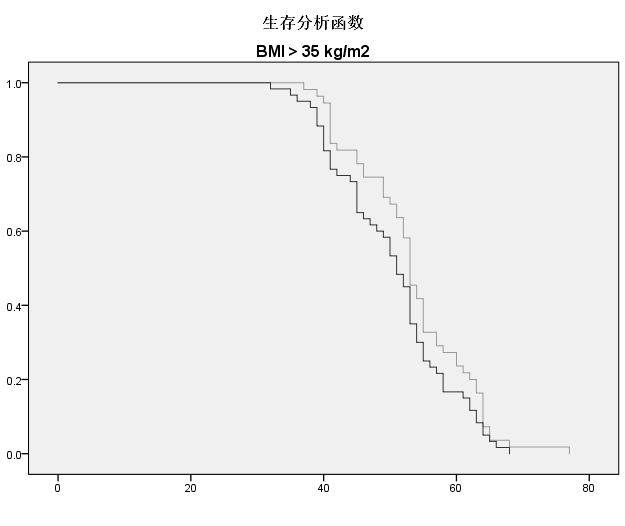

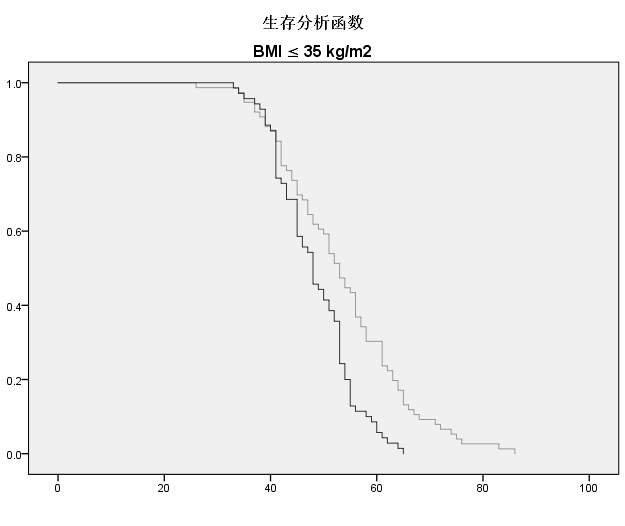


|  | | Median | 95% CI | | Log Rank (*P*) |
| --- | --- | --- | --- | --- | --- |
|  |  |  | lower | upper |  |
| BMI ≤ 35 kg m^-2^ | R group | 53 | 49.898 | 56.102 | 0.001 |
|  | P group | 48 | 44.937 | 51.063 |  |
| BMI > 35 kg m^-2^ | R group | 53 | 51.392 | 54.608 | 0.03 |
|  | P group | 52 | 48.155 | 53.845 |  |

**ASA** (American Society of Anesthesiologists)


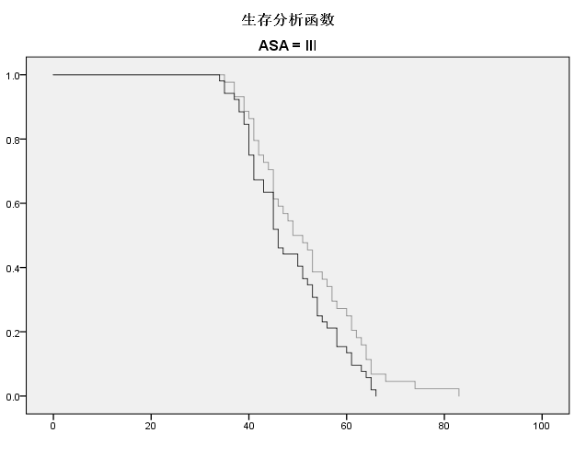

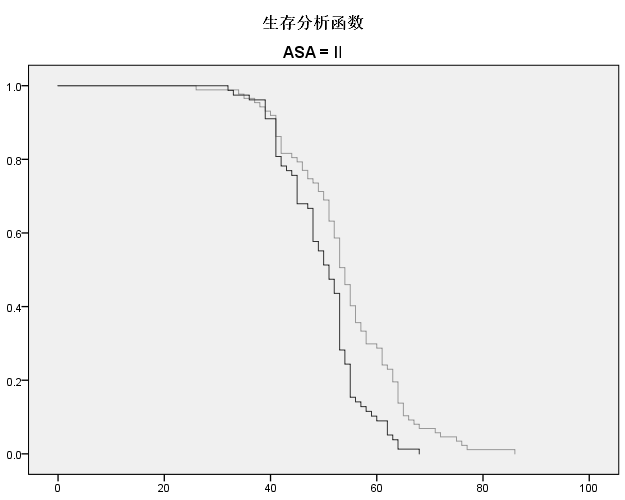


|  | | Median | 95% CI | | Log Rank (*P*) |
| --- | --- | --- | --- | --- | --- |
|  |  |  | lower | upper |  |
| ASA=Ⅱ | R group | 54 | 52.292 | 55.708 | < 0.001 |
|  | P group | 51 | 48.119 | 53,881 |  |
| ASA=Ⅲ | R group | 49 | 44.125 | 53.875 | 0.092 |
|  | P group | 46 | 43.182 | 48.818 |  |

***1.2 Time to recovery (timing unit: min)***

**
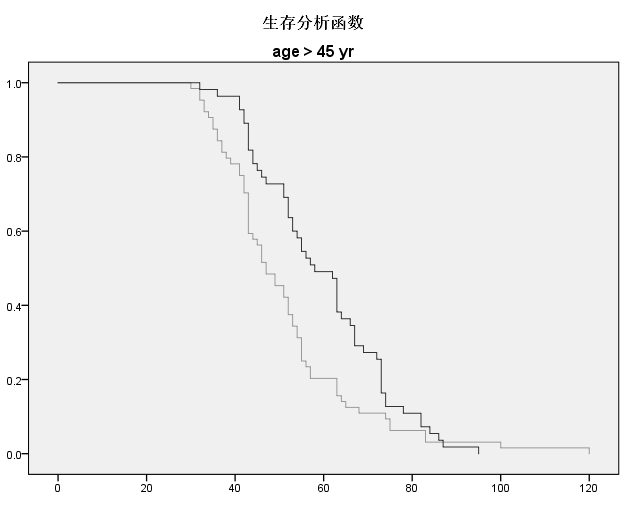
**
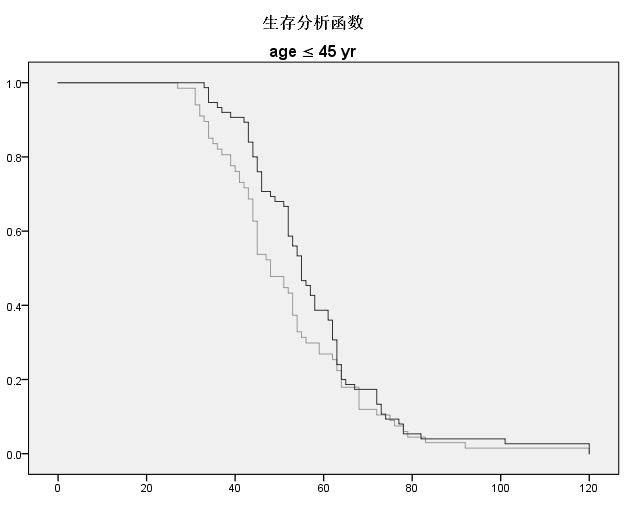
**Age**

|  | | Median | 95% CI | | Log Rank (*P*) |
| --- | --- | --- | --- | --- | --- |
|  |  |  | lower | upper |  |
| Age ≤ 45 yr | R group | 53 | 50.594 | 55.406 | 0.173 |
|  | P group | 48 | 45.32 | 50.68 |  |
| Age > 45 yr | R group | 53 | 51.055 | 54.945 | 0.020 |
|  | P group | 52 | 48.984 | 55.016 |  |

**BMI** (Body mass index)
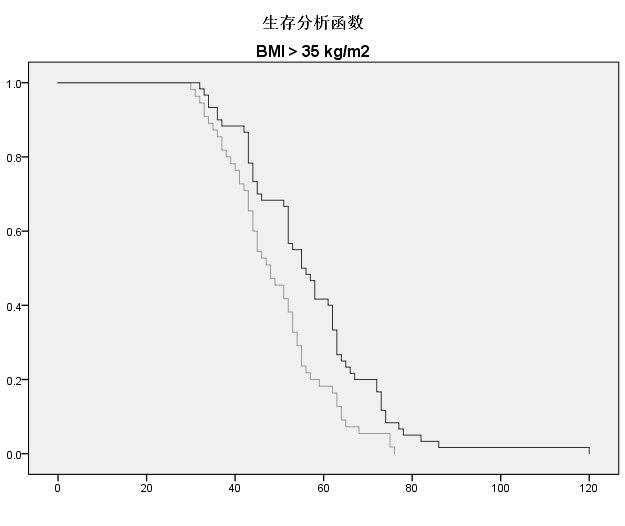


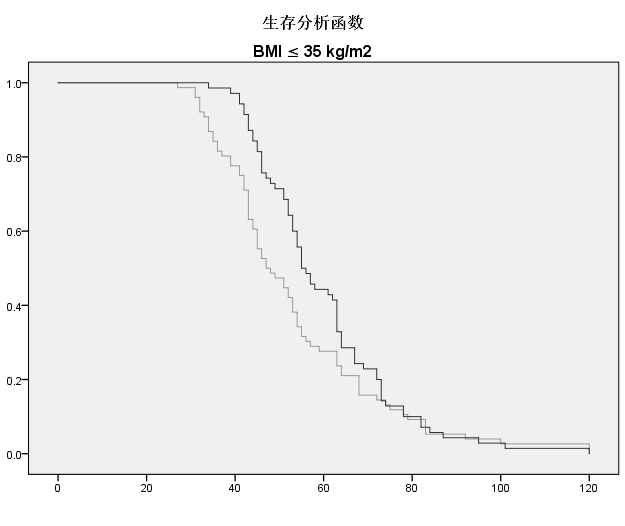


|  | | Median | 95% CI | | Log Rank (*P*) |
| --- | --- | --- | --- | --- | --- |
|  |  |  | lower | upper |  |
| BMI ≤ 35 kg m^-2^ | R group | 48 | 42.065 | 53.935 | 0.149 |
|  | P group | 55 | 50.900 | 59.100 |  |
| BMI > 35 kg m^-2^ | R group | 48 | 42.920 | 53.080 | 0.003 |
|  | P group | 55 | 50.256 | 59.744 |  |

**Gender**


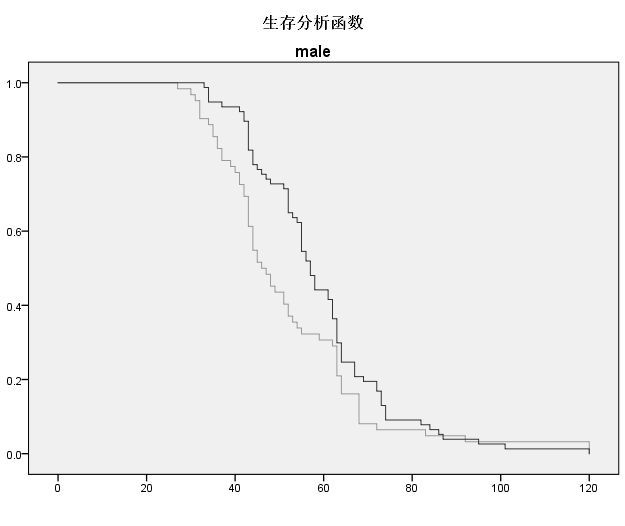

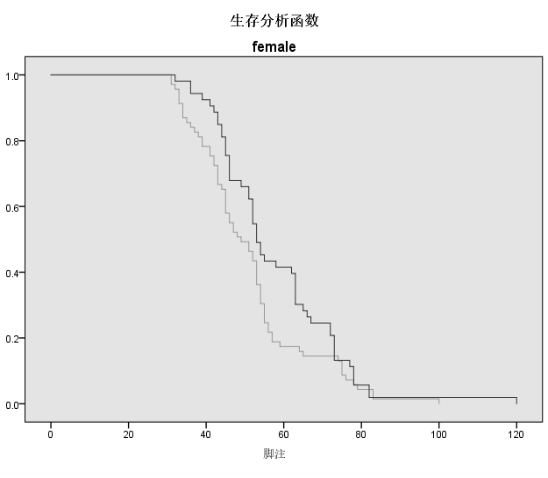


|  | | Median | 95% CI | | Log Rank (*P*) |
| --- | --- | --- | --- | --- | --- |
|  |  |  | lower | upper |  |
| Female | R group | 51 | 44.952 | 55.406 | 0.098 |
|  | P group | 53 | 50.147 | 55.853 |  |
| Male | R group | 46 | 41.791 | 50.209 | 0.065 |
|  | P group | 57 | 54.545 | 59.455 |  |


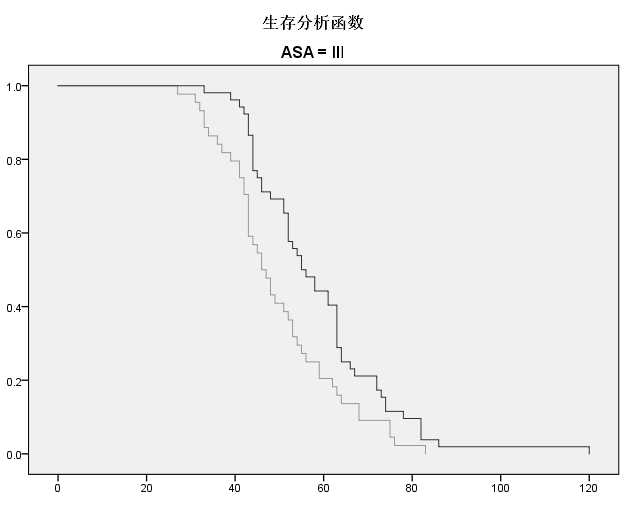
**ASA** (American Society of Anesthesiologists)


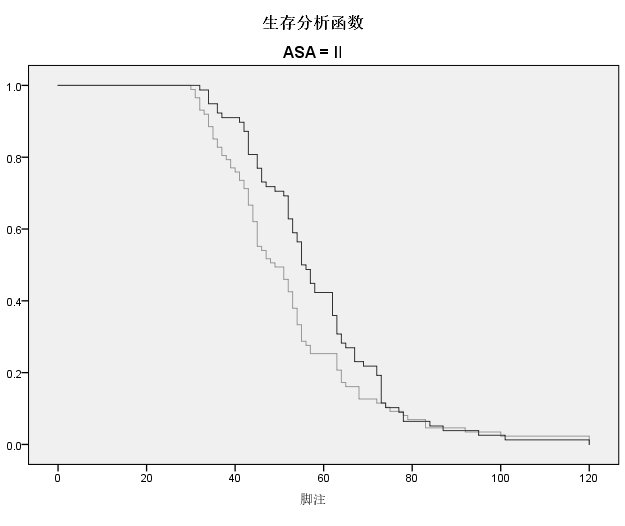


|  | | Median | 95% CI | | Log Rank (*P*) |
| --- | --- | --- | --- | --- | --- |
|  |  |  | lower | upper |  |
| ASA=Ⅱ | R group | 51 | 45.234 | 56.766 | 0.092 |
|  | P group | 55 | 52.115 | 57.885 |  |
| ASA=Ⅲ | R group | 46 | 41.666 | 50.334 | 0.009 |
|  | P group | ;55 | 49.111 | 60.889 |  |

Notes:

Kaplan-Meier survival analysis (stratified test)

1.General binary variables: Age; gender; body mass index (BMI); ASA classification.

2.A two-tailed test *P* < 0.05 was considered statistically significant.

3. Group R : remimazolam + esketamine group ; Group P : propofol + esketamine group.

4.The solid black line represents group P, while the solid gray line represents group R.
