# Supplementary material for: Effects of opioid-free propofol or remimazolam balanced anesthesia on hypoxemia incidence in patients with obesity during gastrointestinal endoscopy: A prospective, randomized clinical trial
Source: Front Med (Lausanne). 2023 Mar 22;10:1124743. doi: 10.3389/fmed.2023.1124743 (PMC10073760; doi:10.3389/fmed.2023.1124743)
Supplement: Supplementary file 2 [file Table_2.docx]

***Supplemental Digital Content 2***

1. **Supplementary Tables**

Type of endoscopy

| Hypoxemia type ^a^ | | *P* | OR | OR 95% CI | |
| --- | --- | --- | --- | --- | --- |
|  |  |  |  | lower | upper |
| Mild hypoxemia | intercept | .350 |  |  |  |
|  | [group R] * [upper gastrointestinal endoscopy] | .155 | 3.077 | .653 | 14.504 |
|  | [group R] * [lower gastrointestinal endoscopy] | .463 | 1.885 | .348 | 10.221 |
|  | [group R] * [combined gastrointestinal endoscopy] | . | . | . | . |
|  | [group P] * [upper gastrointestinal endoscopy] | . | . | . | . |
|  | [group P] * [lower gastrointestinal endoscopy] | . | . | . | . |
|  | [group P] * [combined gastrointestinal endoscopy] | . | . | . | . |
| Severe hypoxemia | intercept | .232 |  |  |  |
|  | [upper gastrointestinal endoscopy] | .084 | .333 | .096 | 1.161 |
|  | [lower gastrointestinal endoscopy] | .831 | .873 | .251 | 3.040 |
|  | [combined gastrointestinal endoscopy] | . | . | . | . |
|  | [group R] * [upper gastrointestinal endoscopy] | .305 | 2.769 | .395 | 19.400 |
|  | [group R] * [lower gastrointestinal endoscopy] | .581 | .529 | .055 | 5.079 |
|  | [group R] * [combined gastrointestinal endoscopy] | . | . | . | . |
|  | [group P] * [upper gastrointestinal endoscopy] | . | . | . | . |
|  | [group P] * [lower gastrointestinal endoscopy] | . | . | . | . |
|  | [group P] * [combined gastrointestinal endoscopy] | . | . | . | . |

Age

| Hypoxemia type^a^ | | *p* | OR | OR 95% CI | |
| --- | --- | --- | --- | --- | --- |
|  |  |  |  | lower | upper |
| Mild hypoxemia | intercept | .003 |  |  |  |
|  | [group R] * [Age ≤ 45 yr  ] | .267 | .515 | .160 | 1.661 |
|  | [group R] * [Age＞45 yr] | . | . | . | . |
|  | [group P] * [Age ≤ 45 yr  ] | . | . | . | . |
|  | [group P] * [Age＞45 yr] | . | . | . | . |
| Severe hypoxemia | intercept | .009 |  |  |  |
|  | [group R] * [Age ≤ 45 yr  ] | .861 | 1.150 | .240 | 5.501 |
|  | [group R] * [Age＞45 yr] | . | . | . | . |
|  | [group P] * [Age ≤ 45 yr  ] | . | . | . | . |
|  | [group P] * [Age＞45 yr] | . | . | . | . |

BMI (Body mass index)

| Hypoxemia type^a^ | | *P* | OR | OR 95% CI | |
| --- | --- | --- | --- | --- | --- |
|  |  |  |  | lower | up |
| Mild hypoxemia | intercept | .021 |  |  |  |
|  | [group R] * [BMI ≤ 35 kg m^-2^] | .486 | .663 | .208 | 2.112 |
|  | [group R] * [BMI ＞ 35 kg m^-2^] | . | . | . | . |
|  | [group P] * [BMI ≤ 35 kg m^-2^] | . | . | . | . |
|  | [group P] * [BMI ＞ 35 kg m^-2^] | . | . | . | . |
| Severe hypoxemia | intercept | .014 |  |  |  |
|  | [group R] * [BMI ≤ 35 kg m^-2^] | .906 | .909 | .188 | 4.393 |
|  | [group R] * [BMI ＞ 35 kg m^-2^] | . | . | . | . |
|  | [group P] * [BMI ≤ 35 kg m^-2^] | . | . | . | . |
|  | [group P] * [BMI ＞ 35 kg m^-2^] | . | . | . | . |

Stop - Bang Score

| Hypoxemia type^a^ | | *P* | OR | OR 95% CI | |
| --- | --- | --- | --- | --- | --- |
|  |  |  |  | lower | upper |
| Mild hypoxemia | intercept | .320 |  |  |  |
|  | [group R] * [Stop - Bang Score ≤ 5] | .917 | 1.065 | .327 | 3.466 |
|  | [group R] * [Stop - Bang Score ＞ 5] | . | . | . | . |
|  | [group P] * [Stop - Bang Score ≤ 5] | . | . | . | . |
|  | [group P] * [Stop - Bang Score ＞ 5] | . | . | . | . |
| Severe hypoxemia | intercept | .517 |  |  |  |
|  | [group R] * [Stop - Bang Score ≤ 5] | .416 | .379 | .037 | 3.916 |
|  | [group R] * [Stop - Bang Score ＞ 5] | . | . | . | . |
|  | [group P] * [Stop - Bang Score ≤ 5] | . | . | . | . |
|  | [group P] * [Stop - Bang Score ＞ 5] | . | . | . | . |

Neck circumference

| Hypoxemia type^a^ | | *P* | OR | OR 95% CI | |
| --- | --- | --- | --- | --- | --- |
|  |  |  |  | lower | up |
| Mild hypoxemia | intercept | .758 |  |  |  |
|  | [group R] * [Neck circumference ≤ 40 cm] | .893 | 1.093 | .300 | 3.985 |
|  | [group R] * [Neck circumference > 40 cm] | . | . | . | . |
|  | [group P] * [Neck circumference ≤ 40 cm] | . | . | . | . |
|  | [group P] * [Neck circumference > 40 cm] | . | . | . | . |
| Severe hypoxemia | intercept | .758 |  |  |  |
|  | [group R] * [Neck circumference ≤ 40 cm] | .901 | 1.181 | .087 | 16.058 |
|  | [group R] * [Neck circumference > 40 cm] | . | . | . | . |
|  | [group P] * [Neck circumference ≤ 40 cm] | . | . | . | . |
|  | [group P] * [Neck circumference > 40 cm] | . | . | . | . |

Ischemic heart illness

| Hypoxemia type^a^ | | *P* | OR | OR 95% CI | |
| --- | --- | --- | --- | --- | --- |
|  |  |  |  | lower | upper |
| Severe hypoxemia | intercept | .079 |  |  |  |
|  | [group R] * [No history of schemic heart illness] | .587 | 1.452 | .378 | 5.580 |
|  | [group R] * [History of schemic heart illness] | . | . | . | . |
|  | [group P] * [No history of schemic heart illness] | . | . | . | . |
|  | [group P] * [History of schemic heart illness] | . | . | . | . |
| Severe hypoxemia | intercept | .591 |  |  |  |
|  | [group R] * [No history of schemic heart illness] | .455 | 1.851 | .368 | 9.315 |
|  | [group R] * [History of schemic heart illness] | . | . | . | . |
|  | [group P] * [No history of schemic heart illness] | . | . | . | . |
|  | [group P] * [History of schemic heart illness] | . | . | . | . |

Gender

| Hypoxemia type ^a^ | | *P* | OR | OR 95% CI | |
| --- | --- | --- | --- | --- | --- |
|  |  |  |  | lower | upper |
| Mild hypoxemia | intercept | .002 |  |  |  |
|  | [group R] * [female] | .827 | 1.143 | .346 | 3.776 |
|  | [group R] * [male] |  |  |  |  |
|  | [group P] * [female] |  |  |  |  |
|  | [group=1] * [male] |  |  |  |  |
| Severe hypoxemia | intercept | .000 |  |  |  |
|  | [group R] * [female] | .338 | .452 | .089 | 2.292 |
|  | [group R] * [male] |  |  |  |  |
|  | [group P] * [female] |  |  |  |  |
|  | [group P] * [male] |  |  |  |  |

Notes:

Interaction (intervention * stratification variables)

1. Group R : remimazolam + esketamine group ; Group P : propofol + esketamine group; Interventions refer to different groups
2. Interactions between intervention and stratification variables were analyzed by multivariate mixed-effect logistic regression models; *P* values were not corrected by Bonferroni method, and <0.05 was considered significantly different.
3. a: The categorical dependent variable was divided into three types, and the reference baseline was “no hypoxemia”.
4. These binary variables were filtered out by the mixed-effects logistic model (*P* < 0.1).
5. The Odd Ratio (OR) values in this table were not adjusted.
