# Supplementary material for: Effects of opioid-free propofol or remimazolam balanced anesthesia on hypoxemia incidence in patients with obesity during gastrointestinal endoscopy: A prospective, randomized clinical trial
Source: Front Med (Lausanne). 2023 Mar 22;10:1124743. doi: 10.3389/fmed.2023.1124743 (PMC10073760; doi:10.3389/fmed.2023.1124743)
Supplement: Supplementary file 3 [file Data_Sheet_1.docx]

***Supplemental Digital Content 3***

**Supplementary Figures**


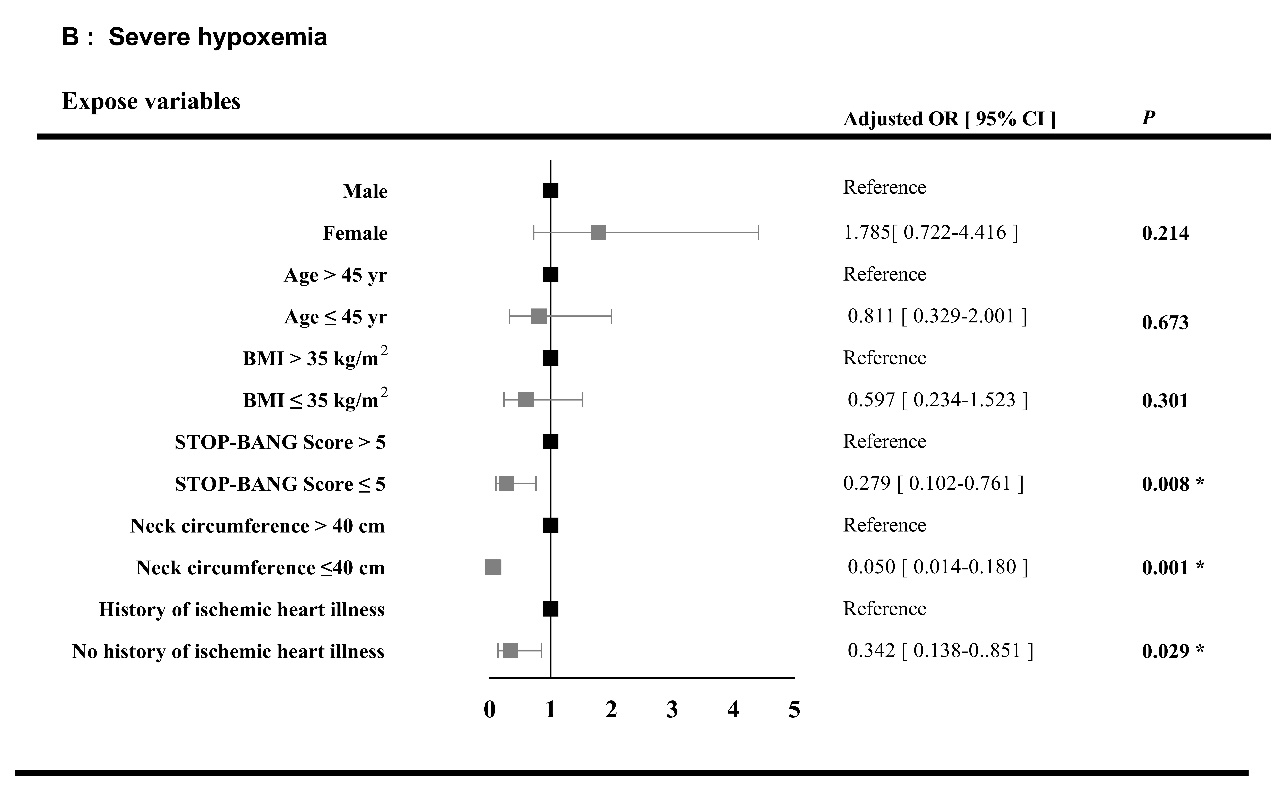

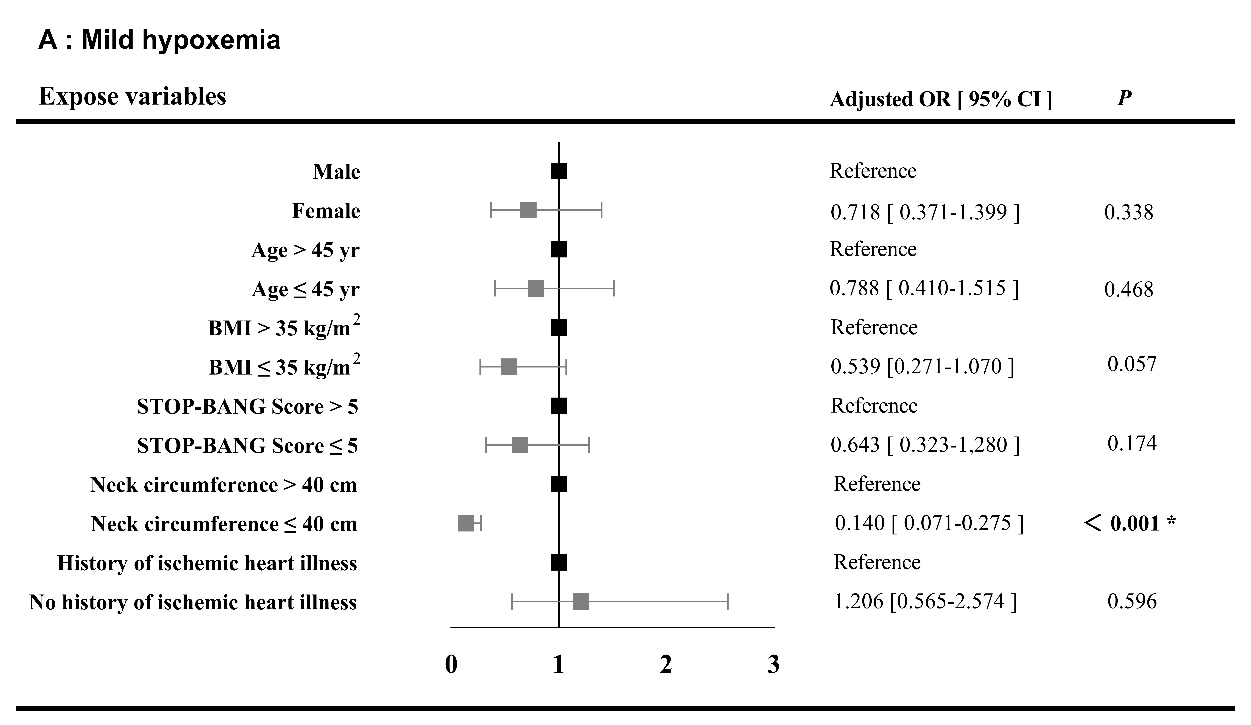
Figure. S3 Forest plot showing multiple mixed effects logistic regression

Notes: 1. Adjusted OR (aOR) and their 95% confidence interval (CI) were obtained by mixed-effect logistic regression and 2000 bootstrapped samples of the study population. 2. *P*-values were not adjusted for multiple testing and *P* < 0.05 was considered statistically significant. 3. “*” represents that this binary variable as an independent factor influencing the occurrence of hypoxemia. 4. BMI (body mass index) is the weight in kilograms divided by the square of the height in meters.
